# Covert Information Mapped Spatial and Directional Modulation toward Secure Wireless Transmission

**DOI:** 10.3390/s21227646

**Published:** 2021-11-17

**Authors:** Jie Tian, Hao Chen, Zhigang Wang, Xianhua Shi, Zhengyu Ji, Xianglu Li

**Affiliations:** 1 Institute of Electronic Engineering, China Academy of Engineering Physics, Mianyang 621999, China; tianjie@caep.cn (J.T.); sonic12345qwert@gmail.com (X.S.); tianliudou@gmail.com (Z.J.); 2 Key Laboratory of Science and Technology on Communications, University of Electronic Science and Technology of China, Chengdu 611731, China; 202021220202@std.uestc.edu.cn; 3 Guangdong Communications and Networks Institute, Guangzhou 510289, China; wangzhigang@gdcni.cn

**Keywords:** spatial and directional modulation (SDM), covert information mapping (CIM), eavesdropping

## Abstract

Recently, the concept of spatial and direction modulation (SDM) has been developed to reap the advantages of both spatial modulation (SM) and directional modulation (DM). On the one hand, DM ensures the transmission security at the expected direction. On the other hand, the structure of SM-aided distributed receivers can enhance the security even if the eavesdropper is located in the same direction as the legitimate receiver. However, the above advantages are achieved based on the assumption that the eavesdropper is not equipped with distributed receivers. On the other hand, the information security can no longer be guaranteed when the eavesdropper is also equipped with distributed receivers. To alleviate this problem, we considered a joint design of SDM and covert information mapping (CIM) in order to conceive of a more robust structure of CIM-SDM. Furthermore, both the detection performances at the eavesdropper and the legitimate user were quantified through theoretical derivation. In general, both the analysis and simulation results supported that the proposed CIM-SDM structure provides more robust secure performance compared to the original SDM, even if the extreme condition of distributed receivers at the eavesdropper is considered, at the cost of moderate performance loss at the legitimate user.

## 1. Introduction

Due to the increase in wireless communications, the pursuit of an extremely high transmission rate [1,2] massive access capability [3], and ultra-low-latency communications (URLLC) [4] has been realized gradually with the development of the Fifth-Generation (5G) and beyond networks. Meanwhile, considering the open transmission environment of wireless communications, especially in device-to-device (D2D) networks, and so on [5], the desire for physical layer security [6] has attracted much attention, in order to protect the communications between the legitimate transmitter and receiver, while preventing an unintended receiver from eavesdropping. The design toward physical layer security includes the reconstruction of the wireless waveform [7], information mapping styles [8], and artificial noise injection [9].

Directional modulation (DM) [10,11,12], as an efficient way to disturb the wireless waveform in the unexpected directions, has attracted much attention to provide the physical security between the transmitter (Alice) and the legitimate receiver (Bob) while degrading the quality of signal detection at the eavesdropper (Eve). Specifically, the idea of directional modulation has been widely considered to adopt multicarrier transmission [13], polarized antennas [14], and the antenna array design [15,16,17,18], aiming at enhancing the information security for the current wireless systems by preventing Eve from eavesdropping. However, the above-mentioned security advantage will no longer exist when Eve takes the opportunity to share the same receive direction as Bob. In this case, the original performance gap between Eve and Bob will shrink due to the loss of protection of the waveform from Alice.

To address this issue, an integration with spatial modulation (SM) [19,20,21,22] has been introduced toward the so-called spatial and directional modulation (SDM) [23] system, in order to prevent eavesdropping. On the one hand, the unique way of information modulation for SM with index modulation [24], utilizing the index of the activated antenna, is combined with DM. On the other hand, distributed receivers are employed at Bob [25], so as to utilize the index of the real activated receiver to deliver information. The SDM system can ensure secure information transmission, on the assumption that Eve does not have distributed receivers. However, in many practical situations, Eve tends to increase the hardware cost in order to enhance the detection quality. In this case, if Eve also adopts distributed receivers and each approaches Bob’s subreceivers, the information security will no longer be upheld.

Recently, the idea of secret modulation has also been considered as an efficient way to protect legitimate information even if the waveform is detected. Specifically, the combination of secret modulation and media-based modulation (MBM) was considered in [26]. The extension to modulation with legitimate channels was explored in [27]. The combination of the channel state information at the transmitter toward secure modulation was considered in [28]. Specifically, a class of novel security transmission techniques is covert information mapping (CIM) [1], toward enhancing the information security even if Eve has received the identical waveform as Bob. The initial idea of CIM was to embed the covert information into the spatial modulation waveform, so as to prevent Eve from demodulating this covert information from a disguised waveform that shares the identical statistics as the original SM waveform. The idea of CIM can be easily extended to current modulation schemes such as OFDM-IM [29] and multiple domain IM [30]. However, to the best of the authors’ knowledge, the combination of CIM and SDM to reap their advantages in a secure waveform and information mapping has not been proposed in the current literature.

Against this background, in this paper, we aim at combing the concept of CIM and SDM, in order to prevent Eve from eavesdropping and hence to provide physical transmission security from the aspects of both waveform design and information modulation. The technique’s contributions can be summarized as follows. Firstly, the basic structure of CIM-SDM is studied with modeling, by combining CIM and SDM in the context of distributed receivers. Furthermore, the detection performances at Bob and Eve are both quantified through theoretical analysis, in order to demonstrate the advantages of the developed CIM-SDM system in terms of secure information transmission. Lastly, the simulation results are exhibited on different parameters, to support the theoretical results and prove that CIM-SDM can effectively avert Eve even if Eve is equipped with the identical distributed resources as Bob, so as to provide enhanced information security.

The remainder of this paper is organized as follows. In Section 2, the system model of the original SDM is analyzed, while the structure of the proposed CIM-SDM system is described in more detail. In Section 3, we derive the average bit error probability (ABEP) upper bound for Bob and Eve and analyze their characteristics, while in Section 4, the simulation results are outlined to demonstrate the advantages of the proposed system. Finally, our conclusions are offered in Section 5.

Notation: In this paper, ${\left(\cdot \right)}^{H}$ represents the conjugate transpose of a matrix, and $∥\cdot ∥$ represents the Frobenius norm. Moreover, $R\left(\cdot \right)$ and CN indicate the real operators and circularly symmetric complex Gaussian distribution, respectively.

## 2. Convert Information Mapping for SDM

**Figure 1:**
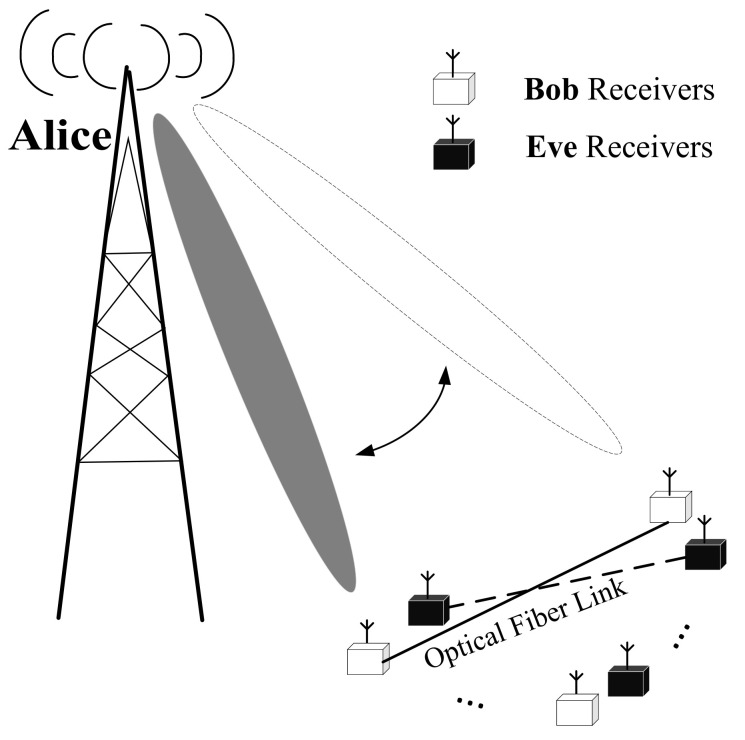
Systematic scenario of directional modulation.

As shown in Figure 1, we considered a directional modulation system where the transmitter, Alice, is equipped with $N_{t}$ antennas and the legitimate user, Bob, is equipped with $N_{r}$ cooperative single-antenna receivers, which connect to each other through optical fibers. We assumed that Alice is capable of obtaining the directions of Bob’s receivers, while the unintended receiver with $N_{e}$ antennas, as Eve, conceals its eavesdropping on the transmit signals. In this section, we first introduce the traditional SDM scheme, and then, the proposed CIM-SDM scheme and its detection algorithm are detailed.

### 2.1. Traditional SDM System Model

For the sake of improving the transmission rate, spatial modulation was introduced to DM by utilizing the index of the receiver to convey additional information [23]. In order to introduce the model more intuitively, we use math equations to describe the principle of the traditional SDM system. More specifically, in SDM, the input bitstreams are partitioned into blocks of $k_{1}+k_{2}$ bits, where the first $k_{1}=\log_{2}N_{r}$ bits are mapped into the index of the activated receiver and the remaining $k_{2}=\log_{2}M$ bits are mapped into the conventional *M*-ary amplitude-phase modulation (APM) symbol. Consequently, the SDM symbol $s_{i}^{j}$ can be expressed as:

(1) $$\begin{array}{c} s_{i}^{j}=e_{i}b_{j}, \end{array}$$

where $e_{i}$ represents the *i*-th column vectors selected from the identify matrix $I_{N_{r}\times N_{r}}$ and *i* denotes the activated receiver index. Furthermore, $b_{j}$, j=1,2,…,M represents the selected *M*-ary APM symbol transmitted toward the activated receiver. Then, a precoding matrix $P=\left[p_{1},p_{2},\ldots ,p_{N_{r}}\right]$ is designed to ensure that only the *i*-th legitimate receiver is activated without power leakage to the remaining receivers, and hence, the transmitted signal vector x is given by:

(2) $$\begin{array}{c} x={\mathrm{Ps}}_{i}^{j}, \end{array}$$

where the *i*-th column vector $p_{i}$ of the precoding matrix P can be formulated as:

(3) $$\begin{array}{c} p_{i}=h\left(\theta_{i}\right)/N_{t}, \end{array}$$

and $h\left(\theta_{i}\right)$ denotes the channel vector between the *i*-th receiver of Bob and Alice. To be more explicit, in this paper, we considered that Alice sets an array geometric center and equips a uniform linear array to generate the directional beam to Bob, and hence, the free space channel vector $h\left(\theta_{i}\right)$ can be detailed as:

(4) $$\begin{array}{c} h\left(\theta_{i}\right)={\left[e^{j\Phi_{0}\left(\theta_{i}\right)},e^{j\Phi_{1}\left(\theta_{i}\right)},\ldots ,e^{j\Phi_{u}\left(\theta_{i}\right)},\ldots ,e^{j\Phi_{N_{t-1}}\left(\theta_{i}\right)}\right]}^{H}, \end{array}$$

with:

(5) $$\begin{array}{c} \Phi_{u}\left(\theta_{i}\right)=-j2\pi \left(\left(N_{t}-1\right)/2-u\right)d\cos\theta_{i}/\lambda , \end{array}$$

where λ is the signal wavelength and d⩽λ/2 represents the antenna spacing.

As a result, the receive signal vector at Bob can be expressed as:

(6) $$\begin{array}{c} y_{B}=H\left(\Theta_{B}\right)x+n_{B}=H\left(\Theta_{B}\right){\mathrm{Ps}}_{i}^{j}+n_{B}, \end{array}$$

with:

(7) $$\begin{array}{c} H\left(\Theta_{B}\right)={\left[h\left(\theta_{1}\right),h\left(\theta_{2}\right),\ldots ,h\left(\theta_{N_{r}}\right)\right]}^{H}, \end{array}$$

where $H\left(\Theta_{B}\right)$ denotes the channel matrix and $\Theta_{B}=\left\{\theta_{1},\theta_{2},\ldots ,\theta_{N_{r}}\right\}$ represents the direction of Bob. Moreover, $n_{B}$ represents additive Gaussian white noise (AWGN) obeying $CN∼\left(0,\sigma_{n}^{2}I_{N_{r}\times N_{r}}\right)$. As for Eve, the receive signal can be formulated as:

(8) $$\begin{array}{c} \begin{array}{cc} y_{E} & =H\left(\Theta_{E}\right)x+n_{E}\\ \; & =H\left(\Theta_{E}\right){\mathrm{Ps}}_{i}^{j}+n_{E}, \end{array} \end{array}$$

where $\Theta_{E}=\left\{\theta_{1},\theta_{2},\ldots ,\theta_{N_{e}}\right\}$ represents the direction of Eve and $H\left(\Theta_{E}\right)$ is the channel matrix of Eve. Furthermore, $n_{E}$ also represents the AWGN obeying $CN∼\left(0,\sigma_{E}^{2}I_{N_{e}\times N_{e}}\right)$. According to (6) and (8), if Eve also has the same number of distributed receivers with similar directions to Bob, Eve may successfully eavesdrop on the signal transmitted from Alice to Bob after carefully observing the activated situation of the receivers. Hence, the security of the transmit signal may be compromised.

### 2.2. Proposed CIM-SDM Scheme

In order to improve the security of the information from the transmitter to the legitimate receiver and further suppress the performance of the unintended receivers, we propose the covert information mapping for spatial and directional modulation (CIM-SDM) scheme, where a special bits-to-symbol mapping regime is carefully designed to conceal spatial index information. It is worth noting that the special bits-to-symbol mapping regime is known to both the transmitter and legitimate user, while Eve hardly notices, which improves the transmission security. The details of the proposed scheme are proposed as follows.

At the transmitter, unlike traditional SDM, the activated receiver index is carefully conceived to hide the real index information, while Eve still deems it to be a conventional spatially modulated antenna index. To elaborate a little further, from the bitstreams’ perspective, a block of $k_{1}+k_{2}+k_{3}$ bits is divided into three parts. First, the indexes of Bob’s distributed receivers are partitioned into two groups, namely $I_{1}$ and $I_{2}$, with $I_{1}=\left\{1,2,\ldots ,\frac{N_{r}}{2}\right\}$ and $I_{2}=\left\{\frac{N_{r}}{2}+1,\frac{N_{r}}{2}+2,\ldots ,N_{r}\right\}$. Then, the first $k_{1}$ bits are utilized to select the receiver group. For example, if $k_{1}=\left[1\right]$, the receiver group index at the *k*-th data block retains the same one as the previous. Otherwise, the receiver group index will be different. According to this special mapping rule, let us assume that the selected receiver group at the (k−1)-th data block is $I_{1}$, i.e., $I_{g_{k-1}}=I_{1}$, and then the receiver group at the *k*-th data block can be obtained as:

(9) $$\begin{array}{c} I_{g_{k}}=\left\{\begin{array}{cc} I_{1}, & \mathrm{if}\;k_{1}=\left[1\right]\;\mathrm{and}\;I_{g_{k-1}}\;=\;I_{1}\;\\ I_{2}, & \mathrm{if}\;k_{1}=\left[0\right]\;\mathrm{and}\;I_{g_{k-1}}\;=\;I_{1} \end{array}\right.. \end{array}$$

Similarly, for the case of $I_{g_{k-1}}=I_{2}$, $I_{g_{k}}$ can be obtained as:

(10) $$\begin{array}{c} I_{g_{k}}=\left\{\begin{array}{cc} I_{2}, & \mathrm{if}\;k_{1}=\left[1\right]\;\mathrm{and}\;I_{g_{k-1}}\;=\;I_{2}\;\\ I_{1}, & \mathrm{if}\;k_{1}=\left[0\right]\;\mathrm{and}\;I_{g_{k-1}}\;=\;I_{2} \end{array}\right.. \end{array}$$

On the basis of determining the receiver group, the following $k_{2}=\log_{2}\left(\frac{N_{r}}{2}\right)$ bits are then applied to choose one receiver index out of the receiver group $I_{g_{k}}$ as $I_{g_{k}}\left(d\right)$. Hence, in CIM-SDM, the index of the activated receiver is different from the index *i* generated by traditional spatial modulation, and the real index information intelligently hides in the switching of the receiving antenna group. This implies that Eve cannot correctly demodulate the transmitter information, since Eve is unaware of the disguised message regime.

Ultimately, the last $k_{3}=\log_{2}M$ bits are mapped into the conventional APM symbol $b_{j}$. The CIM-SDM symbol can be expressed as:

(11) $$\begin{array}{c} s_{I_{g_{k}}\left(d\right)}^{j}=e_{I_{g_{k}}\left(d\right)}b_{j}, \end{array}$$

and the transmitted signal vector is analogous to (2), which can be detailed as:

(12) $$\begin{array}{c} x_{k}={\mathrm{Ps}}_{I_{g_{k}}\left(d\right)}^{j}. \end{array}$$

Then, $x_{k}$ is transmitted to Bob by the directional beam, which is similar to SDM. It is worth noting that CIM-SDM adopts the transmitter architecture of SDM and still utilizes the receiver index to convey additional bit information. However, the modulation from the bit to receiver index is different from the traditional SDM. Hence, the security of the transmission can be improved.

Here, we give a simple example to elaborate on our proposed scheme. First, we assumed that Alice is equipped with four antennas and quadrature phase shift keying (QPSK) modulation, while Bob has four distributed single-antenna receivers. Furthermore, let us denote the receiver group index at the (k−1)-th data block by $I_{g_{k-1}}=I_{1}$, and a 4 bit sequence $b_{k}={\left[0,0,1,1\right]}^{T}$ is considered at the *k*-th data block. On the basis of (9) and $k_{1}=\left[0\right]$, $I_{g_{k}}$ will be different from the previous one, i.e., $I_{g_{k}}=I_{2}$. Next, based on the second bit $k_{2}=\left[0\right]$, the activated receiver index $I_{g_{k}}\left(d\right)=3$ is selected from $I_{2}$ for directional modulation. The remaining bits $k_{3}=\left[1,1\right]$ are mapped into the QPSK symbol $b_{4}=-1+i$. Finally, the transmission signal vector can be expressed as $x_{k}={\mathrm{Ps}}_{I_{g_{k}}\left(k\right)}^{j}={\mathrm{Ps}}_{3}^{4}=Pe_{3}b_{4}$.

**Figure 2:**
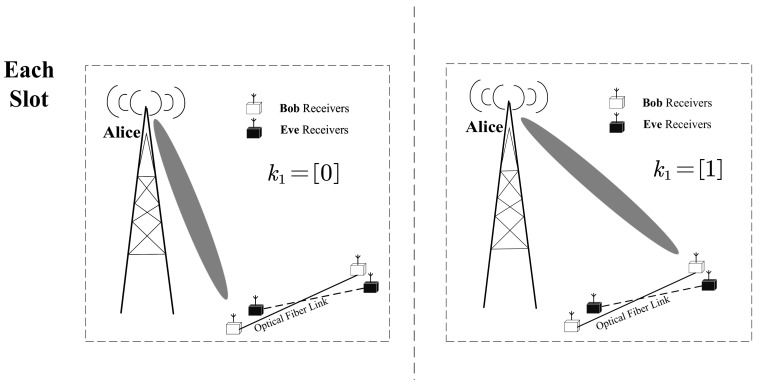
An example with $N_{r}=2$ for the traditional SDM activated process.

Moreover, we considered the basis situation in which Bob has two distributed single-antenna receivers. In order to explain the differences between CIM-SDM and SDM more clearly, we first introduce the activation rule of SDM, as shown in Figure 2. At each slot, SDM independently determines the activated receiver based on the input bits $k_{1}$. More specifically, we assumed that $k_{1}=\left[0\right]$ and $k_{1}=\left[1\right]$ represent the activation of the first and the second receiver, respectively, in the context of SDM.

**Figure 3:**
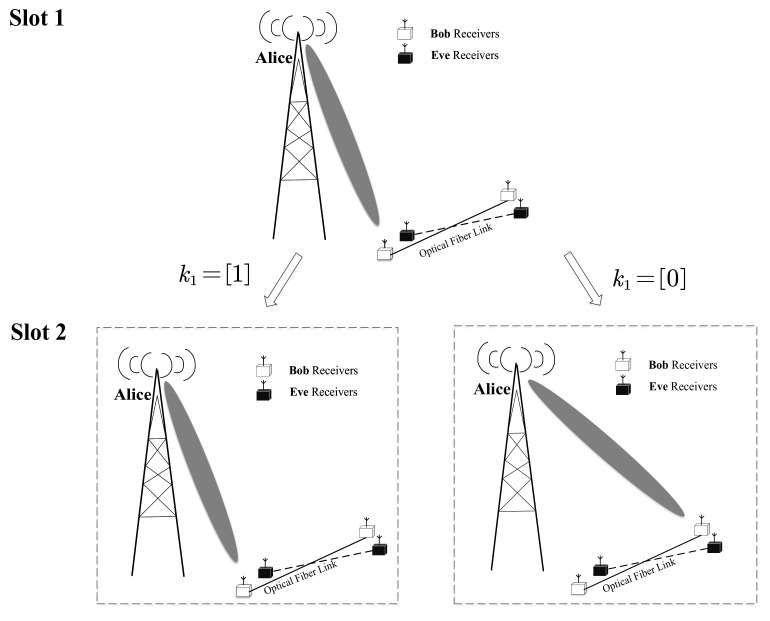
An example with $N_{r}=2$ for the traditional SDM activated process.

However, in CIM-SDM, the activated receiver selection is jointly determined by the previous slot i−1 and the present slot *i*, as shown in Figure 3. For example, we assumed that the initial activated receiver at Slot 1 is the first one. Then, different from the traditional SDM, if $k_{1}=\left[1\right]$, the activated receiver at Slot 2 remains the same as Slot 1, i.e., the activated receiver is the first one. Otherwise, the activated receiver switches into the second one. Based on this special mapping rule, the CIM-SDM scheme successfully disguises itself as a traditional SDM, but having reversed bit information compared to that of SDM. Therefore, Eve with a similar direction decodes the received information incorrectly, which improves the transmission security.

### 2.3. Detection Algorithm for the Proposed Scheme

Similarly, the receive signal vector of Bob at the *k*-th data block can be formulated as:

(13) $$\begin{array}{c} \begin{array}{cc} y_{B_{k}} & =H\left(\Theta_{B}\right)x_{k}+n_{B}\\ \; & =H\left(\Theta_{B}\right){\mathrm{Ps}}_{I_{g_{k}}\left(d\right)}^{j}+n_{B}. \end{array} \end{array}$$

For the sake of simplicity, we used the symbol $i_{c}$ to replace the symbol $I_{g_{k}}\left(d\right)$. On the basis of (13), the optimal detector for Bob jointly considers the activated receiver index $i_{c}$ and the modulated symbol $b_{j}$, yielding:

(14) $$\begin{array}{c} \langle {\hat{i}}_{c},b_{\hat{j}}\rangle =\arg\min_{{\hat{i}}_{c}\in I,b_{\hat{j}}\in B}\left\{{∥y_{B_{k}}-H\left(\Theta_{B}\right){\mathrm{Ps}}_{{\hat{i}}_{c}}^{\hat{j}}∥}^{2}\right\}, \end{array}$$

where $I=\left\{1,2,\ldots ,N_{r}\right\}$ represents the set of the indices of Bob’s receiver and B denotes the constellation of *M*-ary QAM symbols.

After confirming the activated receiver index ${\hat{i}}_{c}$, we determined the receiver group set as:

(15) $$\begin{array}{c} {\hat{g}}_{k}=\left\{\begin{array}{c} 1,\;\mathrm{if}\;{\hat{i}}_{c}⩽\frac{N_{r}}{2}\;\\ 2,\;\mathrm{if}\;{\hat{i}}_{c}>\frac{N_{r}}{2} \end{array}\right.. \end{array}$$

Combined with the (k−1)-th receiver group set ${\hat{g}}_{k-1}$, the bit for receiver group selection can be obtained in comparison to ${\hat{g}}_{k-1}$, which can be detailed as follows,

(16) $$\begin{array}{c} k_{1}=\left\{\begin{array}{cc} 1, & {\hat{g}}_{k}={\hat{g}}_{k-1}\\ 0, & {\hat{g}}_{k}\neq {\hat{g}}_{k-1} \end{array}\right.. \end{array}$$

**Figure 4:**
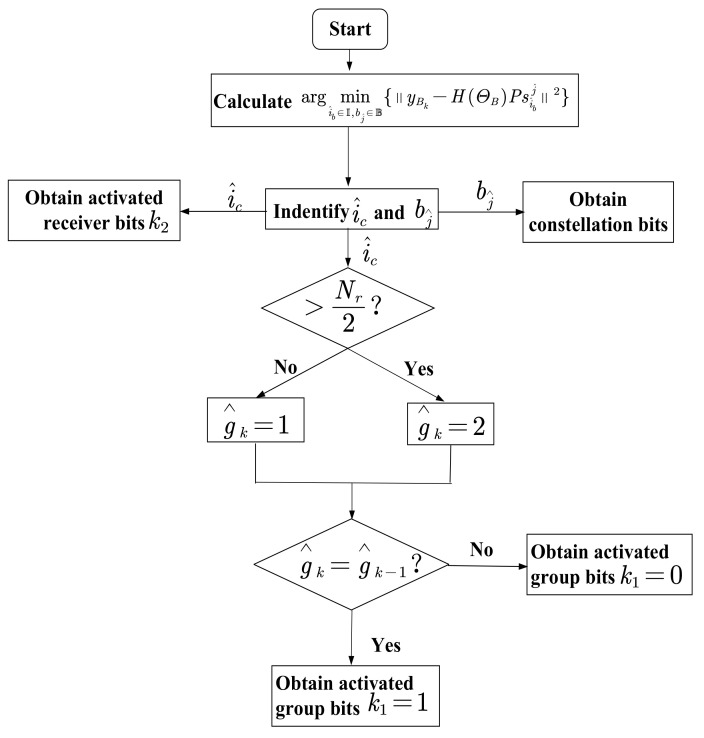
Flowchart depicting Bob’s detection algorithm adopted in the proposed CIM-SDM system.

Moreover, the activated receiver index out of $I_{{\hat{g}}_{k}}$ can be given by:

(17) $$\begin{array}{c} \hat{d}=\mathrm{MO}D_{N_{r}/2}\left({\hat{i}}_{c}-1\right)+1, \end{array}$$

where $\mathrm{MO}D_{a}\left(b\right)$ returns the remainder after division of *b* by *a*. Finally, $\hat{d}$ is capable of being demapped into the receiver index bits. Figure 4 depicts a flowchart of the aforementioned detection algorithm.

As for Eve, the receive signal vector of Eve at the *k*-th data block can be expressed as:

(18) $$\begin{array}{c} \begin{array}{cc} y_{E_{k}} & =H\left(\Theta_{E}\right)x_{k}+n_{E}\\ \; & =H\left(\Theta_{E}\right){\mathrm{Ps}}_{I_{g_{k}}\left(d\right)}^{j}+n_{E}; \end{array} \end{array}$$

however, it is difficult to recognize whether a covert transmission has occurred even if the channel state information and beamforming matrix can be obtained. More specifically, under the assumption that Eve successfully eavesdrops on the transmitted signal, the optimal ML detection for the transmitted signal in the context of Eve can be given by:

(19) $$\begin{array}{c} \langle {\hat{i}}_{c},b_{\hat{j}}\rangle =\arg\min_{{\hat{i}}_{c}\in I,b_{\hat{j}}\in B}\left\{{∥y_{E_{k}}-H\left(\Theta_{E}\right){\mathrm{Ps}}_{{\hat{i}}_{c}}^{\hat{j}}∥}^{2}\right\}. \end{array}$$

However, since Eve is unaware of the covert information mapping scheme, ${\hat{i}}_{c}$ and $b_{\hat{j}}$ are then directly demapped from the bits, which leads to an undesirable decoding performance.

Owing to its peculiar bits-to-symbol mapping regime, the proposed CIM-SDM scheme is able to conceal the real bit information to preclude Eve from decoding it without a transmission rate decrease, which leads to a promising secure transmission technique.

## 3. Performance Analysis

In this section, the average bit error probability (ABEP) union bound of Bob and Eve is analyzed and derived when communicating over free space channels under the hypothesis of an optimal joint detector being invoked. These derivations are based on the union bound technique described in [31].

### 3.1. Bob’s Average Bit Error Probability

More specifically, at Bob, let the set of all possible CIM-SDM symbols be denoted by S and the ABEP of the proposed CIM-SDM scheme at *k*-th data block be upper bounded by:

(20) $$\begin{array}{c} P_{B_{k}}⩽\frac{1}{R_{p}2^{R_{p}}}\sum_{s_{i_{c}}^{j}\in S}^{}\sum_{s_{m_{c}}^{n}\in S\neq s_{i_{c}}^{j}}^{}d\left(s_{i_{c}}^{j}\to s_{m_{c}}^{n}\right)P\left(s_{i_{c}}^{j}\to s_{m_{c}}^{n}\right), \end{array}$$

where $R_{p}$ denotes the transmission rate of the proposed scheme, i.e., $R_{p}=\log_{2}N_{r}+\log_{2}M(bpcu)$, which is the same as in the traditional SDM. Moreover, $d\left(s_{i_{c}}^{j}\to s_{m_{c}}^{n}\right)$ represents the Hamming distance between the equivalent bit representations of $s_{i_{c}}^{j}$ and $s_{m_{c}}^{n}$, and $P\left(s_{i_{c}}^{j}\to s_{m_{c}}^{n}\right)$ denotes the pairwise error probability (PEP).

In particular, the PEP in (20) can be formulated as:

(21) $$\begin{array}{c} \begin{array}{c} P\left(s_{i_{c}}^{j}\to s_{m_{c}}^{n}\right)\\ =P\left(∥y_{B_{k}}-H_{\Lambda }s_{i_{c}}^{j}∥>∥y_{B_{k}}-H_{\Lambda }s_{m_{c}}^{n}∥\right)\\ =P\left(∥H_{\Lambda }s_{i_{c}}^{j}+n_{B}-H_{\Lambda }s_{i_{c}}^{j}∥>∥H_{\Lambda }s_{i_{c}}^{j}+n_{B}-H_{\Lambda }s_{m_{c}}^{n}∥\right)\\ =P\left(0>{∥H_{\Lambda }s_{i_{c}}^{j}-H_{\Lambda }s_{m_{c}}^{n}∥}^{2}+2R\left[{\left(H_{\Lambda }s_{i_{c}}^{j}-H_{\Lambda }s_{m_{c}}^{n}\right)}^{H}n_{B}\right]\right)\\ =P(-R\left[{\left(H_{\Lambda }s_{i_{c}}^{j}-H_{\Lambda }s_{m_{c}}^{n}\right)}^{H}n_{B}\right]>{∥H_{\Lambda }s_{i_{c}}^{j}-H_{\Lambda }s_{m_{c}}^{n}∥}^{2}/2). \end{array} \end{array}$$

where we define $H_{\Lambda }=H\left(\Theta_{B}\right)P$.

Since $n_{B}$ represents the AWGN vector obeying $CN∼\left(0,\sigma_{n}^{2}I_{N_{r}\times N_{r}}\right)$, we can derive that $-R\left[{\left(H_{\Lambda }s_{i_{c}}^{j}-H_{\Lambda }s_{m_{c}}^{n}\right)}^{H}n_{B}\right]$ is a Gaussian random variable obeying CN∼$\left(0,\frac{1}{2}\sigma_{n}^{2}{∥H_{\Lambda }\left(s_{i_{c}}^{j}-s_{m_{c}}^{n}\right)∥}^{2}\right)$. Therefore, the PEP $P\left(s_{i_{c}}^{j}\to s_{m_{c}}^{n}\right)$ can be further given by:

(22) $$\begin{array}{c} \begin{array}{c} P\left(s_{i_{c}}^{j}\to s_{m_{c}}^{n}\right)\\ =Q\left(\sqrt{\frac{{∥H_{\Lambda }\left(s_{i_{c}}^{j}-s_{m_{c}}^{n}\right)∥}^{2}}{2\sigma_{n}^{2}}}\right). \end{array} \end{array}$$

Finally, by substituting (22) into (20), we obtain the upper bound of Bob’s ABEP at the *k*-th data block. However, we introduced the covert information mapping regime, and the bit demapping is associated with the previous data block, i.e., the (k−1)-th data block. Based on this regime, the ABEP of the proposed CIM-SDM scheme entails jointly considering the previous and the present data blocks.

More specifically, the covert information regime has no influence on the PEP and the Hamming distance between $s_{i_{c}}^{j}$ and $s_{m_{c}}^{n}$, but affects the mapping from the embedded bits to the original bits. For example, if the receiver group at the (k−1)-th data block is misjudged, the original bit information at the *k*-th data block may not be obtained correctly, according to (16). Hence, the average error probability of the bit for receiver group selection can be approximated by:

(23) $$\begin{array}{c} \begin{array}{c} P_{r}=P_{B_{k-1}}\times \left(1-P_{B_{k}}\right)+\left(1-P_{B_{k-1}}\right)\times P_{B_{k}}, \end{array} \end{array}$$

which implies that the misjudgment of the receiver group at the (k−1)-th or *k*-th data block results in the misjudgment of the original bit, even though the remaining data block is correctly demodulated. Using $P_{B_{k-1}}=P_{B_{k}}$, Equation (23) can be further simplified as:

(24) $$\begin{array}{c} P_{r}=2P_{B_{k}}\left(1-P_{B_{k}}\right). \end{array}$$

Then, since the bit error probability associated with receiver group selection has been jointly considered, the ABEP of the (k−1)-th and *k*-th data blocks also entails being jointly calculated, which can be updated as:

(25) $$\begin{array}{c} \begin{array}{cc} {\bar{P}}_{B} & =\frac{1}{\log_{2}N_{r}M}\times P_{r}+\frac{\left(\log_{2}N_{r}M-1\right)}{\log_{2}N_{r}M}\times P_{B_{k}}\\ \; & =\frac{\left(\log_{2}N_{r}M+1-2P_{B_{k}}\right)}{\log_{2}N_{r}M}P_{B_{k}}. \end{array} \end{array}$$

If $P_{B_{k}}$ is far less than $log_{2}N_{r}M+1$, Equation (25) can be further approximated by:

(26) $$\begin{array}{c} {\bar{P}}_{B}\approx \left(1+\frac{1}{\log_{2}N_{r}M}\right)P_{B_{k}}. \end{array}$$

Finally, we obtain the ABEP ${\bar{P}}_{B}$ of Bob.

### 3.2. Eve’s Average Bit Error Probability

Based on the optimal joint detector, at Eve, the ABEP of the proposed CIM-SDM scheme at the *k*-th data block can be upper bounded by:

(27) $$\begin{array}{c} P_{E_{k}}⩽\frac{1}{R_{p}2^{R_{p}}}\sum_{s_{i_{c}}^{j}\in S}^{}\sum_{s_{m_{c}}^{n}\in S\neq s_{i_{c}}^{j}}^{}d\left(s_{i_{c}}^{j}\to s_{m_{c}}^{n}\right)P_{H\left(\Theta_{E}\right)}\left(s_{i_{c}}^{j}\to s_{m_{c}}^{n}\right), \end{array}$$

where $P_{H\left(\Theta_{E}\right)}\left(s_{i_{c}}^{j}\to s_{m_{c}}^{n}\right)$ represents the PEP of Eve for a given channel matrix of Even $H\left(\Theta_{E}\right)$. Specifically, the PEP is given by:

(28) $$\begin{array}{c} P_{H\left(\Theta_{E}\right)}\left(s_{i_{c}}^{j}\to s_{m_{c}}^{n}\right)\\ =P\left(∥y_{E_{k}}-{\mathrm{Gs}}_{i_{c}}^{j}∥>∥y_{E_{k}}-{\mathrm{Gs}}_{m_{c}}^{n}∥\right)\\ =P\left(∥{\mathrm{Gs}}_{i_{c}}^{j}+n_{E}-{\mathrm{Gs}}_{i_{c}}^{j}∥>∥{\mathrm{Gs}}_{i_{c}}^{j}+n_{E}-{\mathrm{Gs}}_{m_{c}}^{n}∥\right)\\ =P(-R\left[{\left({\mathrm{Gs}}_{i_{c}}^{j}-{\mathrm{Gs}}_{m_{c}}^{n}\right)}^{H}n_{B}\right]>{∥{\mathrm{Gs}}_{i_{c}}^{j}-{\mathrm{Gs}}_{m_{c}}^{n}∥}^{2}/2), \end{array}$$

where $G=H\left(\Theta_{E}\right)P$. Moreover, $-R\left[{\left({\mathrm{Gs}}_{i_{c}}^{j}-{\mathrm{Gs}}_{m_{c}}^{n}\right)}^{H}n_{B}\right]$ is a Gaussian random variable obeying $CN∼\left(0,\frac{1}{2}\sigma_{E}^{2}{∥G\left(s_{i_{c}}^{j}-s_{m_{c}}^{n}\right)∥}^{2}\right)$. Hence, the PEP can be rewritten as:

(29) $$\begin{array}{c} \begin{array}{c} P_{H\left(\Theta_{E}\right)}\left(s_{i_{c}}^{j}\to s_{m_{c}}^{n}\right)\\ =Q\left(\sqrt{\frac{{∥G\left(s_{i_{c}}^{j}-s_{m_{c}}^{n}\right)∥}^{2}}{2\sigma_{E}^{2}}}\right). \end{array} \end{array}$$

By substituting (29) into (27), the ABEP of the CIM-SDM scheme at the *k*-th data block can be obtained. However, since Eve is unaware of the covert information regime, the correct receiver group information and its corresponding original bit information cannot be obtained. Hence, the average error probability of the bit for receiver group selection can be approximated by $P_{r}=0.5$.

Similarly, the ABEP of the (k−1)-th and *k*-th data block can be formulated as:

(30) $$\begin{array}{c} \begin{array}{cc} {\bar{P}}_{r} & =\frac{1}{\log_{2}N_{e}M}\times P_{r}+\frac{\left(\log_{2}N_{e}M-1\right)}{\log_{2}N_{e}M}\times P_{r_{k}}\\ \; & =\frac{0.5+\left(\log_{2}N_{e}M-1\right)P_{r_{k}}}{\log_{2}N_{e}M}. \end{array} \end{array}$$

Ultimately, the ABEP ${\bar{P}}_{r}$ of Eve is obtained.

## 4. Performance Evaluation

### 4.1. The Implementation of the Simulation Model

**Figure 5:**
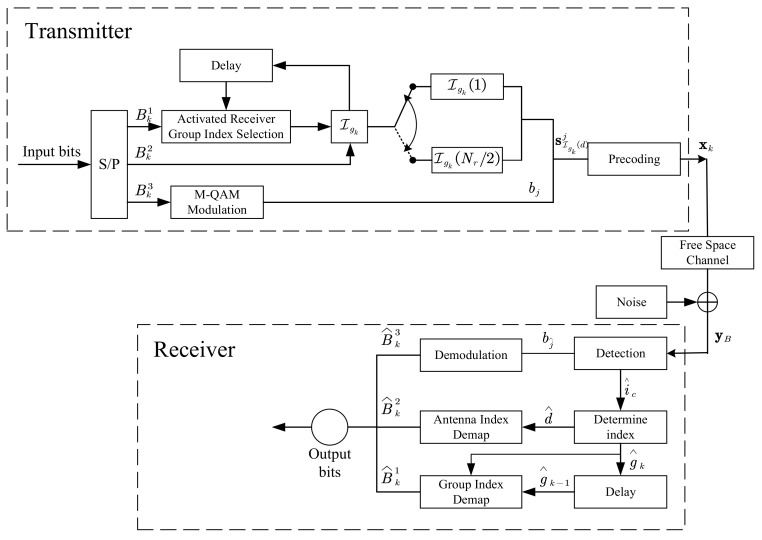
The transceiver architecture of the proposed CIM-SDM scheme.

Based on our proposed CIM-SDM scheme, we first set up the transceiver architecture, as shown in Figure 5. Such an architecture facilitates the implementation of the scheme on a given platform. Then, the proposed CIM-SDM scheme was implemented in MATLAB.

More specifically, we first generated bitstreams. Next, the bitstreams were divided into several parts and mapped into the constellation symbols and activated receiver index. This process simulated the transmitter in real communication scenarios. Then, we generated a channel matrix based on the free space channel model, as we mentioned in Section 2.1, to simulate the channel environment in real communication scenarios. The noise was also considered in our simulated model. Moreover, we conceived of a receiver to detect the signal on the basis of the algorithm proposed in Section 2.3. Finally, the performance of the proposed scheme was evaluated by means of the bit error rate (BER), which can be calculated by comparing the output bits with the input bits.

### 4.2. Simulation Results

In this subsection, we provide the simulation results to characterize the CIM-SDM scheme considered, to illustrate its improved transmission security compared with the traditional SDM. More specifically, we illustrate the system performance against the BER, constellation error rate, and receiver index error rate, under the assumption that Eve has the same number of distributed receivers with similar directions as Bob. Moreover, we assumed that the antenna spacing *d* is λ/4, while other system parameters were as summarized in Table 1.

**Figure 6:**
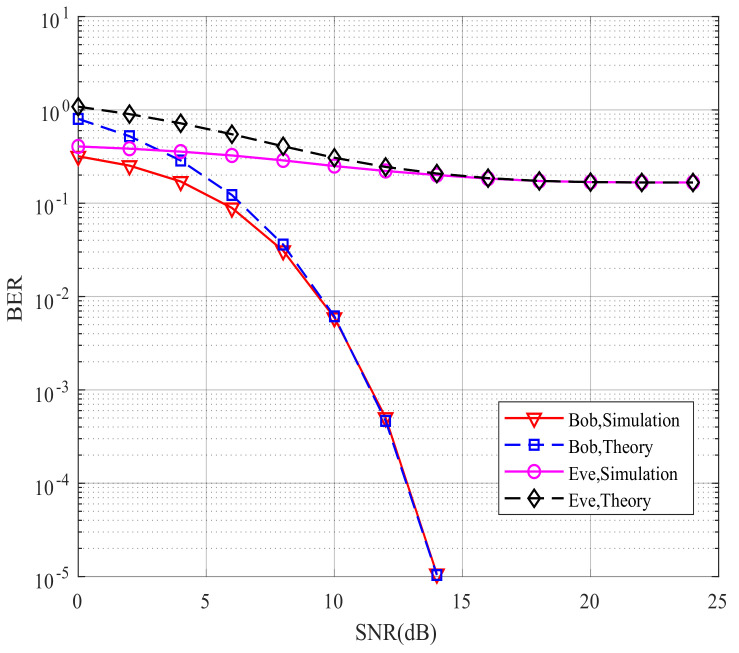
Bob’s and Eve’s theoretical and simulated BER performance for the proposed CIM-SDM scheme employing $N_{t}=8$, $N_{r}=2$, and QPSK.

**Figure 7:**
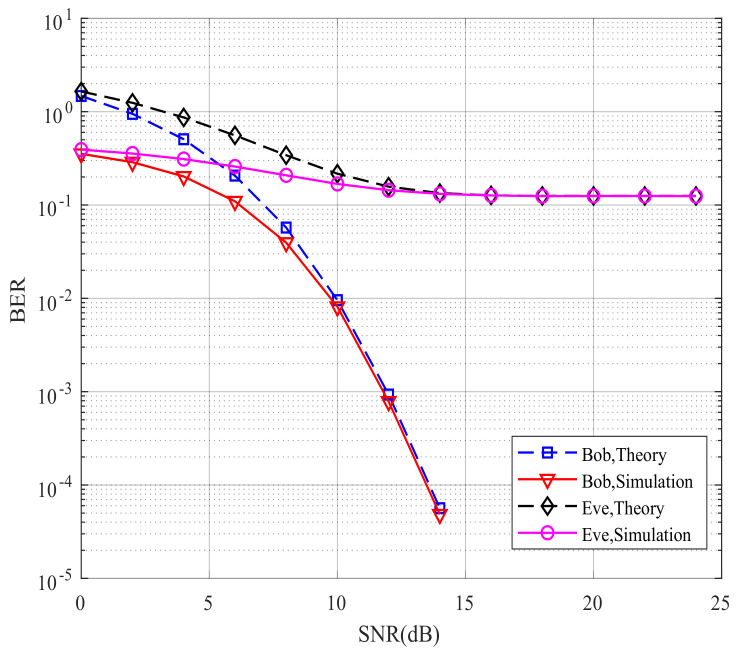
Bob’s and Eve’s theoretical and simulated BER performance for the proposed CIM-SDM scheme employing $N_{t}=8$, $N_{r}=4$, and QPSK.

Figure 6 compares Bob’s and Eve’s theoretical and simulated BER performance for the proposed CIM-SDM scheme in the context of $N_{r}=N_{e}=2$. If the theoretical curves provide a good approximation to the simulated ones, we can validate the effectiveness of the theoretical analysis and use the theoretical analysis to evaluate the system performance in future work. By comparing the curves in Figure 6, we made the following observations. First, in CIM-SDM, the theoretical curves of Bob and Eve form a tight upper bound of the simulated curves at a high signal-to-noise ratio (SNR). Meanwhile, the theoretical curves of Bob and Eve are reachable as the SNR increases. Similar trends are also valid for Figure 7, where $N_{r}=N_{e}=4$, which further validates the effectiveness of the theoretical analysis of (26) and (30).

However, the theory and simulation results differed at a low SNR, as shown in Figure 6 and Figure 7. The reason for this trend is that the union bound theory in [28] has some limitations, since the roles played by the spatial and signal constellation diagrams (and the related bit mapping) are hidden in the four-fold summation. Hence, at a high SNR, the theoretical curve provides a tight upper bound. However, at a low SNR, due to the severe influence of noise and the limitation of the analysis method, the theoretical curve slightly deviates from the corresponding simulation results. However, the theoretical curve is still the upper bound for the simulation result.

**Figure 8:**
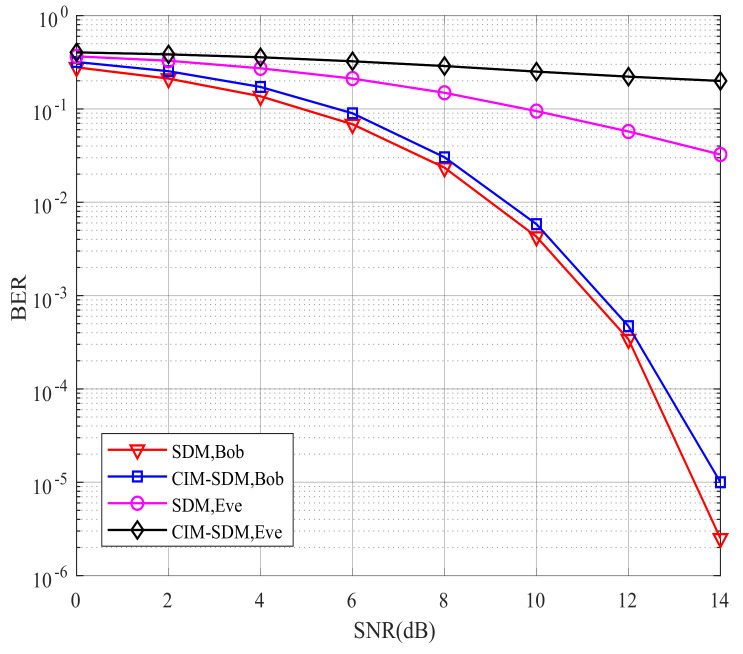
Bob’s and Eve’s BER performances for the proposed CIM-SDM scheme in comparison to the traditional SDM counterparts employing $N_{t}=8$, $N_{r}=2$, and QPSK.

**Figure 9:**
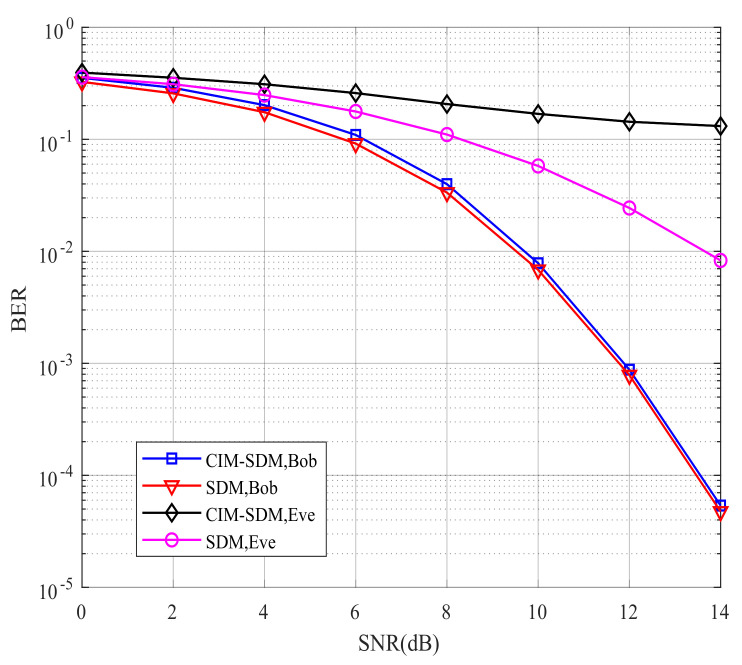
Bob’s and Eve’s BER performances for the proposed CIM-SDM scheme in comparison to the traditional SDM counterparts employing $N_{t}=8$, $N_{r}=4$, and QPSK.

Having validated our theoretical analysis using Figure 6 and Figure 7, we further illustrate the BER performance of the proposed CIM-SDM scheme compared with the traditional SDM scheme in Figure 8 and Figure 9. As shown in Figure 8, for Eve, the proposed CIM-SDM system provides worse BER performance than the SDM scheme. More specifically, in CIM-SDM, Eve approximately exhibited a 6 dB performance loss at a BER of 10^−1^ compared with the traditional SDM. What is more, the BER performance of Eve almost stayed at 0.5, which means that it was difficult for Eve to decode the information correctly in the context of CIM-SDM. Another important observation inferred from Figure 8 is that the CIM-SDM scheme exhibited slight performance loss compared to that of SDM. The reason for this degradation was that the original bit demodulation was associated with the previous and present data block, as we analyzed in (23) and (24).

However, observe from Figure 9 that, as expected, the CIM-SDM scheme approximately achieved the same BER performance as the SDM scheme at Bob when the number of receivers increased. The reason for this trend was that the influence of joint detection error was decreasing, as we analyzed in (26). Moreover, we also note in Figure 9 that the SDM scheme without the covert information mapping regime provided a significantly improved BER performance at Eve with increased receivers, while our CIM-SDM scheme still led to a poor BER performance at almost 10^−1^. Hence, due to the introduction of the special bits-to-symbol mapping regime, our proposed CIM-SDM scheme is capable of increasing the BER performance gap between Bob and Eve, while keeping the same transmission rate as SDM.

**Figure 10:**
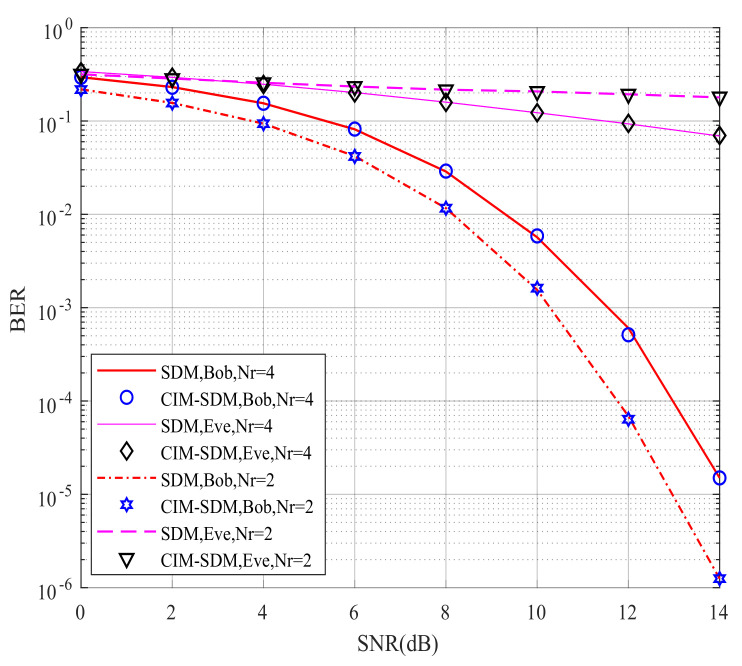
Bob’s and Eve’s constellation error rate performances for the proposed CIM-SDM scheme in comparison to the traditional SDM counterparts.

**Figure 11:**
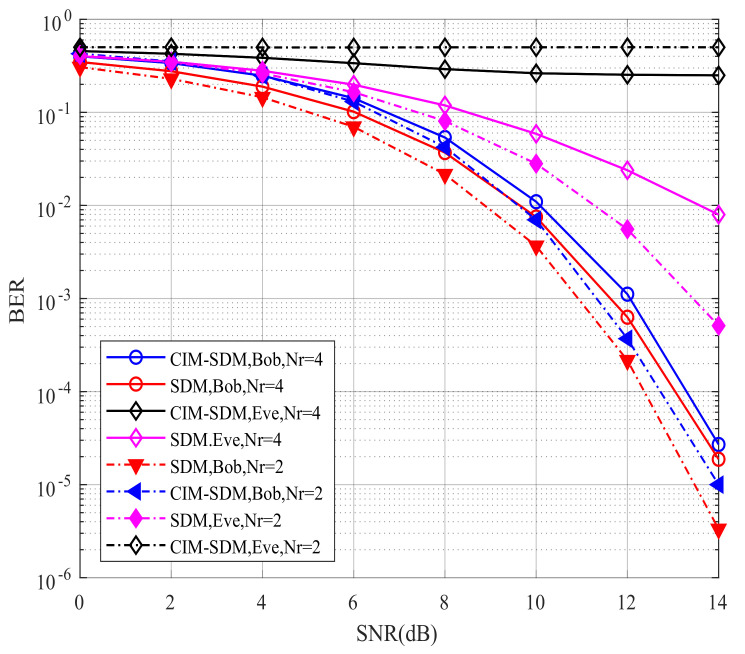
Bob’s and Eve’s receiver index error rate performances for the proposed CIM-SDM scheme in comparison to the traditional SDM counterparts.

In order to further characterize the advantages of the covert information mapping regime, we plot the constellation error rate and receiver index error rate for CIM-SDM and SDM in Figure 10 and Figure 11. In Figure 10, from Bob’s constellation demodulation perspective, the constellation error rate performance of the proposed CIM-SDM scheme achieved the same performance as the traditional SDM in the context of $N_{r}=2$ and $N_{r}=4$. The reason for this trend was that the special space–time mapping regime disguised the activated receiver index instead of the constellation symbols. Hence, the introduction of the space–time mapping regime had no influence on the demodulation of the constellation symbols. On the other hand, at Eve, since the constellation symbols transmitting from Alice to Bob were not disguised, the performance of CIM-SDM remained the same as SDM. This trend further validated the importance of embedded information in secure transmission.

As observed in Figure 11, at Eve, the receiver index error rate performance of CIM-SDM almost remained at 0.5 and suffered significant degradation compared with SDM in the context of $N_{r}=2$, which means Eve was incapable of detecting the receiver index information correctly. The improved security was due to its embedded index, since Eve still considered it as a conventional spatially modulated antenna index. Moreover, in Bob, we notice that the proposed CIM-SDM scheme approximately achieved the same performance as the SDM scheme and exhibited a 0.4 dB performance loss compared to that of SDM at a BER of 10^−4^. A similar trend can be found in the context of $N_{r}=4$. Therefore, the proposed CIM-SDM scheme is preferable to prevent the eavesdropping of Eve at the cost of a slight performance loss at Bob.

## 5. Conclusions

In this paper, the idea of CIM was combined with SDM systems in order to reap their advantages and hence to further improve the transmission security. The combined performance of this system was investigated using computer simulations based on the proposed transceiver architecture.

Our simulation results demonstrated that, due to the special bits-to-symbol mapping regime, the developed CIM-SDM scheme was capable of outperforming the original SDM in preventing Eve from decoding the information correctly, while keeping the same transmission rate as SDM. Even if the eavesdropper is equipped with distributed receivers with identical receive directions to the legitimate user, the proposed CIM-SDM is able to maintain the index information security. Hence, our proposed CIM-SDM scheme may be a promising secure transmission technology.

However, further study is required in order to determine its effectiveness in hardware platforms, in the context of a more realistic environment. For example, in order to realize the directional communication between Alice and Bob in a realistic environment, we will explore the uniform linear array technique, which is capable of changing the beam direction of the antenna system by controlling the phase excitation of the transmitted signal [32,33,34]. Furthermore, to the best of the authors’ knowledge, the uniform linear array has a limited beam scanning range, and hence, the distributed location of Bob should be taken into consideration. Another challenge that may be encountered in the hardware implementation is cooperative communication [35,36,37,38]. It is of paramount importance to devise a cooperative communication framework with high interactivity and quick response among Bob’s receivers. Moreover, this paper only considered the simple free channel model and AWGN environment, and other forms of impairment such as imperfect angle estimation and the multipath effect were not considered. These issues will comprise the topic of our future work.

Additionally, the developed structure has the potential to adopt high-frequency communications [39] with informant protection or further be combined with another covert information transmission scheme in SDM [40], namely spatial and directional modulation with scrambling (SDM-S), to further improve the transmission security. To sum up, the combination of the proposed structure with other promising technologies will also be investigated in the future.

## Figures and Tables

**Table 1:** System parameters.

Figure	Scheme	$N_{t}$	$N_{r}$	*M*	$\Theta_{B}$	$N_{e}$	$\Theta_{E}$
1	CIM-SDM	8	2	4	{15°, 85°}	2	{20°, 60°}
2	CIM-SDM	8	4	4	{15°, 85°, 120°, 210°}	4	{20°, 60°, 110°, 230°}
3	CIM-SDM	8	2	4	{15°, 85°}	2	{20°, 60°}
	SDM	8	2	4	{15°, 85°}	2	{20°, 60°}
4	CIM-SDM	8	4	4	{15°, 85°, 120°, 210°}	4	{20°, 60°, 110°, 230°}
	SDM	8	4	4	{15°, 85°, 120°, 210°}	4	{20°, 60°, 110°, 230°}
5, 6	CIM-SDM	10	2,4	4	{15°, 85°}, {15°, 85°, 120°, 210°}	2,4	{20°, 60°}, {20°, 60°, 110°, 230°}
	SDM	10	2,4	4	{15°, 85°}, {15°, 85°, 120°, 210°}	2,4	{20°, 60°}, {20°, 60°, 110°, 230°}

## Data Availability

Not applicable.
